# 958. Impact of Preexisting Atopic Diseases on Hypertrophic Scarring Outcomes After Burns

**DOI:** 10.1093/jbcr/irag033.536

**Published:** 2026-04-06

**Authors:** Philong Nguyen, Yousef Tanas, Joshua Wang, Kamryn K Baker, Noelle Gonzalez, Sudhanvan Iyer, Gabriela Villa, Hanson Chan, Jong Lee

**Affiliations:** University of Texas Medical Branch, Galveston, TX; Houston Methodist Hospital, Houston, TX; University of Texas Medical Branch, Galveston, TX; McGovern Medical School at University of Texas Health Science Center at Houston, Houston, TX; University of Texas Medical Branch - John Sealy School of Medicine, Houston, TX; University of Texas Medical Branch, Galveston, TX; University of Texas Medical Branch, Galveston, TX; University of Texas Medical Branch, Galveston, TX; Division of Burn and Trauma Surgery, Department of Surgery, University of Texas Medical Branch, Galveston, Galveston, TX

## Abstract

**Introduction:**

Atopic disease has been associated with impaired burn wound healing through barrier dysfunction, chronic inflammation, and immune dysregulation. This disrupted healing may increase susceptibility to hypertrophic scarring, yet the relationship has not been well defined. To date, no known study has specifically examined this relationship. This retrospective cohort study evaluated the association between preexisting atopic disease and hypertrophic scarring after burn injury.

**Methods:**

The TriNetX Research Network was queried to identify patients ages 18 years and older with burn injuries between 2010 and 2024. Two cohorts were defined: patients with a diagnosis of atopic disease (asthma, allergic rhinitis, atopic dermatitis, or eczema) within one year prior to injury, and patients without these conditions. Patients with prior steroid use were excluded. Propensity score matching (1:1) was performed to control for demographics, body mass index, comorbidities, substance use history, and total body surface area burned. Outcomes included hypertrophic scarring at 3 and 12 months. Cox proportional hazards models were used to calculate hazard ratios (HR) with 95% confidence intervals (CI), and statistical significance was set at *p*<.05. Cumulative incidence was reported for each cohort at both time points.

**Results:**

After matching, each cohort included 9660 patients. At 3 months, patients with preexisting atopic disease had higher rates of hypertrophic scarring than non-atopic controls (4.75% vs 3.68%, HR 1.31, 95% CI 1.11–1.55, *p*=.001). By 12 months, this difference persisted and further widened (7.27% vs 5.31%, HR 1.38, 95% CI 1.20–1.58, *p*<.001).

**Conclusions:**

Preexisting atopic disease is associated with a significantly increased risk of hypertrophic scarring after burn injury, underscoring the need for early surveillance and targeted scar management. Prospective studies should confirm causality and evaluate preventive interventions.

**Applicability of Research to Practice:**

Patients with atopic disease face an increased risk of hypertrophic scarring and fibrosis. Clinicians should recognize this vulnerability, initiate early scar-prevention measures, and provide enhanced surveillance and counseling to improve outcomes.

**Funding for the study:**

This study was supported by university funding through the Institute for Translational Sciences (UL1 TR001439), funded by the National Center for Advancing Translational Sciences at the NIH.

